# GC-MS metabolites profiling of anethole-rich oils by different extraction techniques: antioxidant, cytotoxicity and *in-silico* enzymes inhibitory insights

**DOI:** 10.1080/14756366.2022.2097445

**Published:** 2022-07-18

**Authors:** Dina M. El-Kersh, Nada M. Mostafa, Shaimaa Fayez, Tarfah Al-Warhi, Mohammed A. S. Abourehab, Wagdy M. Eldehna, Mohamed A. Salem

**Affiliations:** a Department of Pharmacognosy, Faculty of Pharmacy, The British University in Egypt (BUE), Cairo, Egypt; b Department of Pharmacognosy, Faculty of Pharmacy, Ain-Shams University, Cairo, Egypt; c Department of Chemistry, College of Science, Princess Nourah bint Abdulrahman University, Riyadh, Saudi Arabia; d Department of Pharmaceutics Faculty of Pharmacy, Umm Al-Qura University, Makkah, Saudi Arabia; e Department of Pharmaceutical Chemistry, Faculty of Pharmacy, Kafrelsheikh University, Kafrelsheikh, Egypt; f School of Biotechnology, Badr University in Cairo, Badr City, Cairo, Egypt; g Department of Pharmacognosy and Natural products, Faculty of Pharmacy, Menoufia University, Menoufia, Egypt

**Keywords:** Essential oils, GC-MS profiling, *in-silico* study, metabolomics, antioxidant, cytotoxicity activity

## Abstract

GC-MS profiling and metabolomics study of anise and star anise oils obtained by hydrodistillation, *n*-hexane, and microwave-assisted extraction methods were conducted herein. *Trans*-anethole was the major phenylpropanoid in both oils. Principal component and hierarchical cluster analyses revealed a clear separation of different extraction methods. Microwave-assisted star anise oil (MSA) revealed the highest anethole content (93.78%). MSA oil showed antioxidant activity using DPPH and ABTS assays, this was verified via an *in-silico* docking study of its major compounds on human tyrosinase and NAD(P)H oxidase. *Trans*-anethole displayed the best fitting scores (−8.9 and −10.1 Kcal/mole, respectively). MSA oil showed promising cytotoxic activity on different cell lines, mainly the cervical (HeLa) cell lines. Cell cycle inhibition at the G0–G1 phase was observed with an early apoptotic effect of the oil on HeLa cells. *Trans*-anethole achieved the best docking scores (−7.9, −9.3 and −9.9 Kcal/mole) for *in-silico* study on EGFR, CDK2 and CDK4 enzymes engaged in cancer, respectively.

## Graphical Abstract

**Figure:**
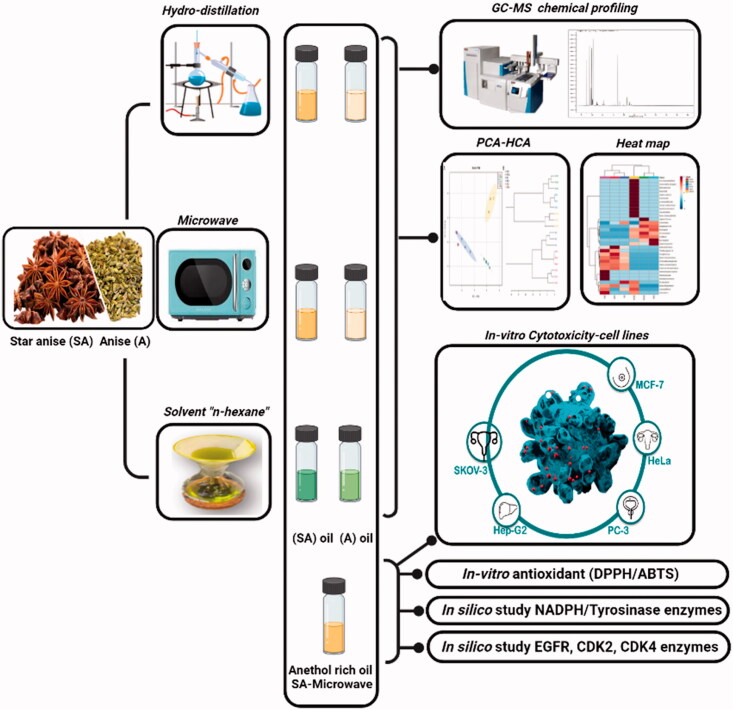

## 1. Introduction

Essential oils (EO) extracted from fruits of aromatic medicinal plants *viz.* anise (*Pimpinella anisum* L., F. Apiaceae) and star anise (*Illicium verum* Hook., F. Schisandraceae) have been used in the pharmaceutical and food industries. For the latter purpose, several reports hinted at their strong antimicrobial, antifungal, and antioxidant efficacies, hence used as preservatives against food-borne pathogens^1^^,^^2^.

In the pharmaceutical field, the essential oils are generally regarded as safe and have been approved by the FDA for their use in the management of flatulence, muscle spasm, and colic, however common adulterants like Japanese star anise fruits (*Illicium anisatum*) are not edible due to reported neurotoxicity^3^. Star anise has likewise been reported to display strong antiviral activity^4^. It is also an industrial source of shikimic acid, the precursor of the anti-avian flu (H5N1 strain) medication oseltamivir (Tamiflu^®^)^4^. For that reason, metabolite profiling of star anise can be of great importance in its identification and quality control analysis.

Several studies were performed to investigate the chemical profile of anise and star anise oils with the latter being rich in phenylpropanoids, monoterpenes, oxygenated monoterpenes, sesquiterpene hydrocarbons, and their oxygenated derivatives^5^. *trans-*Anethole represents the major constituent accounting for the aroma of the oil and reaching in many circumstances up to 90% of the total oil metabolome^6^.

Anise oil is similarly rich in *trans-*anethole (75%–90%) and other constituents including coumarin (i.e. umbelliferone), sterols, and some flavonoids^7^. In general, several factors were reported to affect the yield percentage of oil constituents, among them the extraction process^8^. In Anise oil, *trans-*anethole varied as per the extraction procedures implemented. This in turn affects the overall biological activity since the *n*-hexane extract of star anise displayed more significant antimicrobial effect than that obtained by steam distillation^9^^,^^10^. Furthermore, the oil extracted with ethanol assisted by microwaves showed stronger antioxidant activity than the one extracted by the same solvent in Soxhlet^7^.

The current study aimed to compare the effect of three different extraction procedures *viz.* hydro-distillation, *n*-hexane, and microwave-assisted extraction on both anise and star anise volatiles metabolome. Hierarchical cluster analysis (HCA) and principal component analysis (PCA) were performed with the aim of comparison between the different extraction methods of anise and star anise samples. The overall yield of *trans-*anethole, as well as other constituents of EO of anise and star anise with the impact of the extraction procedures, were assessed via GC-MS analysis. The richest anethole oil was further investigated for its *in vitro* antioxidant and cytotoxicity activities on different cell lines. *In-silico* study of the major chemical constituents of the tested anethole-rich oil was implemented to test the inhibition of NAD(P)H and tyrosinase enzymes. Further, the cytotoxicity mechanism of action was studied with *in silico* modelling of the major compounds of the anethole-rich oil to understand their influence on the cytotoxicity pathways. Interestingly, *trans-*anethole-rich oil displayed promising antioxidant and cytotoxic activities. To the best of our knowledge, this is the first comparative metabolomic study describing the impact of the three different extraction techniques on the yield percentage of *trans-*anethole in both anise and star anise EO.

## 2. Experimental section

### 2.1. Plant materials and extraction of the volatile oils

About 4.5 kg of each anise and star anise dried entire fruits have been purchased from one of the commercial herbal stores in Cairo, Egypt. Both fruits were authenticated by Eng. Therese Labib, Consultant of Plant Taxonomy at the Egyptian Ministry of Agriculture. Voucher specimens of *P. anisum,* F. Apiaceae – anise (PHG-P-PA-360) and *I. verum,* F. Schisandraceae – star anise (PHG-P-IV-361) have been deposited at Pharmacognosy Department, Faculty of Pharmacy, Ain-Shams University. Each anise and star anise dried grinded fruits was subjected individually to oil extraction by three different methods where the extraction per each oil (anise or star anise) per each method of extraction was carried out in three independent experiments. Hydrodistillation: each dried fruits (500 g) was subjected to hydrodistillation (750 ml) using the Clevenger apparatus for 6 h. Oil yields were 4.20 ± 0.20 ml/500 g of anise and 5.20 ± 0.26 ml/500 g of star anise fruits. Solvent extraction: both fruits were extracted by *n*-hexane using 500 g of each fruit cold macerated in 750 ml *n*-hexane (Sigma Aldrich) for 3 days and repeated 3 times till sampling exhaustion. The pooled extracts were concentrated under vacuum to yield 2.33 ± 0.31 and 2.30 ± 0.20 ml of anise and star anise oils, respectively. Microwave-assisted extraction (MAE): 500 g of each fruit was placed in 750 ml distilled water and placed in the microwave for 35 min. at radiation 80% to yield 4.20 ± 0.26 and 6.30 ± 0.26 ml of each anise and star anise oils; respectively. The obtained oils of both fruits of the three different extraction methods were desiccated and then stored in well-sealed opaque vials at 4 °C for further analysis.

### 2.2. The GC-MS analysis of the volatile oils

All oil samples (1 µl injection volume, 1% v/v) were analysed on a Shimadzu GCMS-QP 2010 (Kyoto, Japan) system coupled to a mass spectrometer (SSQ 7000 quadrupole; Thermo- Finnigan, Bremen, Germany) according to the methodology of El-Nashar et al.^11^ and Al-Sayed et al.^12^. The used column for separation of volatile components was RTX-5MS fused bonded column with specifications as follows (30 m × 0.25 mm i.d. × 0.25 μm film thickness). The initial oven temperature was kept at 50 °C for 3 min (isothermal) then programmed to reach 300 °C at 5 °C/minute for 5 min. Both the injector and detector temperatures were fixed at 280 °C, respectively. Helium was used as the carrier gas at a flow rate of 1.41 ml/min. A splitting ratio of 1:15 was employed. The mass spectra were recorded as per the following conditions: filament emission current was 60 mA, 70 eV was the ionisation voltage whereas the ion source was 220 °C. Identification of components was employed as per their Kovat index (KI) as well as their mass spectra where those data were compared to NIST, Adams^13^ and other literature data^14^^,^^15^. The KIs were calculated respective to a series of *n*-alkanes C_8_–C_28_ injected under the same GC conditions and compared to those data published in the literature. Each peak represents a volatile component, whereas its area is calculated as the relative percentage of the whole chromatogram area (100%).

### 2.3. Antioxidant activity in-vitro using DPPH and ABTS assays

Star anise oil was prepared in serial dilutions (15–40 mg/mL) while Trolox standard stock solution (100 µM) was prepared (5–40 µM) in DPPH and ABTS assays; respectively. The DPPH “2,2-diphenyl-1-picryl-hydrazyl-hydrate” and ABTS “(2,2′-azino-bis (3-ethylbenzothiazoline-6-sulfonic acid)) protocols procedures were assessed as per Boly et al.^16^ and Arnao et al.^17^; respectively. Inhibition of DPPH and ABTS colour intensities were assessed through colorimetry at 540 and 734 nm; respectively. The IC_50_ results were presented as means ± SD using microplate reader FluoStar Omega whereas the results were employed using the following equation:

% inhibition = ((Average blank absorbance−Average absorbance of sample)/(Average blank absorbance)) × 100.

Upon calculating antioxidant results in Trolox equivelents (μM Trolox equivalent (TE)/mg star anise oil), the following equations were adopted: *y* = 1.9422x–2.0985 (*R*^2^ = 0.9985) and *y* = 2.3057x–3.97 (*R*^2^ = 0.9996); in DPPH and ABTS; respectively.

### 2.4. Cancer cell lines and cytotoxicity assay using SRB

Cancer cell lines as hepatic (HepG2), ovarian (SKOV-3), breast (MCF-7), cervical (HeLa), and prostate (PC-3) were all obtained from Nawah Scientific Inc. (Mokatam, Cairo, Egypt: nawah-scientific.com). Cells were cultured in: Dulbecco's modified Eagle's media (DMEM) for HepG2, MCF7 and PC-3 and (RPMI) media for SKOV-3 and HeLa cells. A 100 mg/mL streptomycin, 100 units of penicillin as well as 10% of heat-inactivated foetal bovine serum (Sigma Aldrich) in humidified 5% (vol/vol) CO_2_ at a temperature of 37 °C was added to either DMEM or RPMI media as per the type of used cells.

The viability of cells was assessed by SRB (sulphorhodamine B-Sigma Aldrich) assay according to the methodology of Skehan et al.^18^ and Allam et al.^19^. A microplate reader (BMG- LABTECH®-FLUO star Omega, Ortenberg, Germany) was used to measure the absorbance at 540 nm.

### 2.5. Analysis of cell cycle distribution and annexin V-FITC apoptosis assay on HeLa cell line

Both analysis of cell cycle distribution and apoptosis assay methods were adopted from the previous study^20^. Paclitaxel (1 µM) and doxorubicin (10 µM) were used as positive controls in cell cycle analysis distribution and apoptosis assay, respectively. The MSA oil and positive controls were added to HeLa cell lines for 48 h. In apoptosis assay, apoptosis (cell necrosis) was determined using Annexin V-FITC apoptosis detection kit (Abcam Inc., Cambridge Science Park, Cambridge, UK) which is coupled with 2 fluorescent channels flowcytometry. Flow cytometry (ACEA Novocyte™ flow cytometer, ACEA Biosciences Inc., San Diego, CA, USA) was used with ACEA NovoExpress™ software. The results of both cell cycle analysis and apoptosis assessment were expressed as means ± SD.

### 2.6. In silico study of star anise volatiles on human tyrosinase, NAD(P)H oxidase, EGFR, CDK2 and CDK4

The X-ray 3 D structures of human tyrosinase and (NAD(P)H) oxidase were downloaded from the protein data bank using the following IDs: 5m8q^21^ and 2cdu^22^, respectively. The X-ray 3 D structures of EGFR, CDK2 and CDK4 were downloaded from the protein data bank using the following IDs: 1xkk, 1di8 and 2w96, respectively. All the docking studies were conducted using MOE 2019^23^^,^^24^, which was also used to generate the 2D interaction diagrams between the docked ligands and their potential targets. In the beginning, the five enzymes, co-crystalized ligands, and the three identified volatiles (*trans*-anethole, estragole and D-limonene) were prepared using the default parameters. The active site of each target was determined from the binding of the corresponding co-crystalized ligand. After that, the three volatiles were saved into a single file with an MDB extension. Finally, the docking was finalised by docking the MDB file containing the three ligands into the active site of the five enzymes.

### 2.7. Statistical and multivariate analysis

The GC data sets were imported into Microsoft Excel (Excel 2010, Microsoft^®^, Redmond, USA). The percentage composition of each of the six oil samples was presented as mean value ± SD where the statistical analyses were done using the algorithms embedded within Microsoft excel. IC_50_ (% inhibition) of antioxidant and cytotoxicity studies were calculated using GraphPad Prism 6.01, CA, USA. In antioxidant assays, the concentrations were converted to their log values then selected of non-linear inhibitor regression equation (log (inhibitor) vs. normalised response – variable slope equation).

Multivariate data analysis (MVA) was conducted using Metaboanalyst 3.0 software^25^. For Metaboanalyst parameters, the following settings were used: sample normalisation, none; data scaling, Pareto-scaled (mean-centered values/square root of the standard deviation); and data transformation, log transformation. For statistical significance, one-way ANOVA was selected using Bonferroni correction for *p* values. PCA and HCA were used to present the unsupervised clustering pattern of anise and star anise essential oil composition, prepared by different extraction methods. For hierarchical clustering, distance measures using Euclidean distances and clustering algorithms using Ward's linkage were chosen. Clustering results were shown as heatmap and dendrogram.

## 3. Results and discussion

### 3.1. GC-MS analysis of anise and star anise oils by different extraction techniques

Plants are usually rich in bioactive phytochemicals to which the biological effects are mainly attributed^26–30^. In the current study, all the tested oil samples revealed *trans*-anethole as the major component and it was observed that the best extraction methodology was the one assisted by microwaves (MAE), this came in accordance with previous data reported by Cai et al.^9^ Estragole (the structural isomer of *trans*-anethole) was detected in star anise oil by all the applied means of extraction in a ratio reaching 3.44% of the oil, it was only detected in minor concentrations (*ca.* 0.16%) in anise oil obtained solely by solvent extraction.

**Figure 1.**
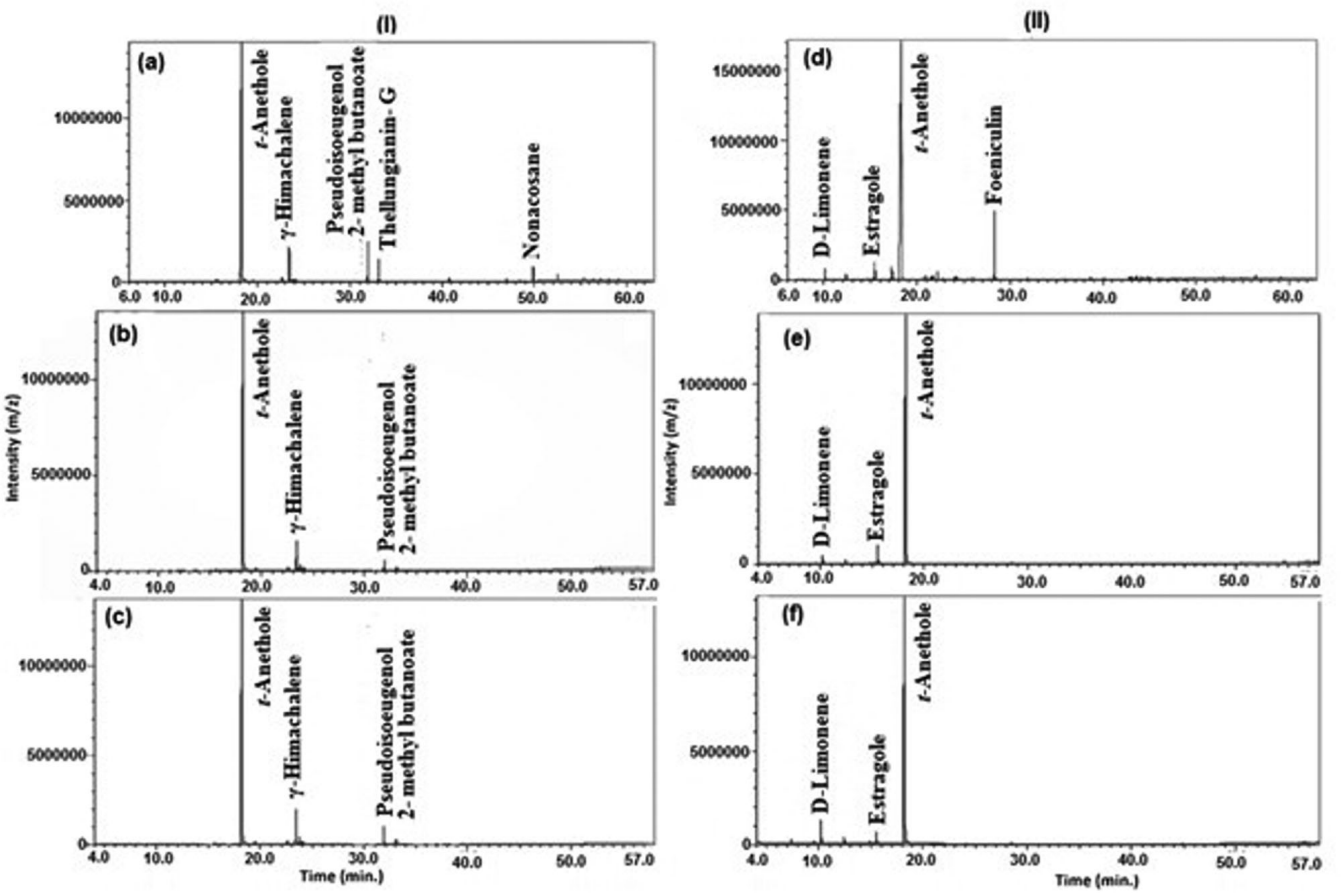
GC-MS analysis of volatile oils of (I) anise and (II) star anise extracted by different techniques, (a, d) solvent extraction, (b, e) microwave-assisted extraction, and (c, f): hydro-distillation, showing the predominance of *trans-*anethole in both oils.

The GC-MS analysis of all EO showed different classes of volatile organic compounds. The privilege of phenyl propanoid class in all extraction procedures with percentile ranging from (88.25 to 97.22%) exemplified in *trans-*anethole has been observed as in (Figure 1).

**Figure 2.**
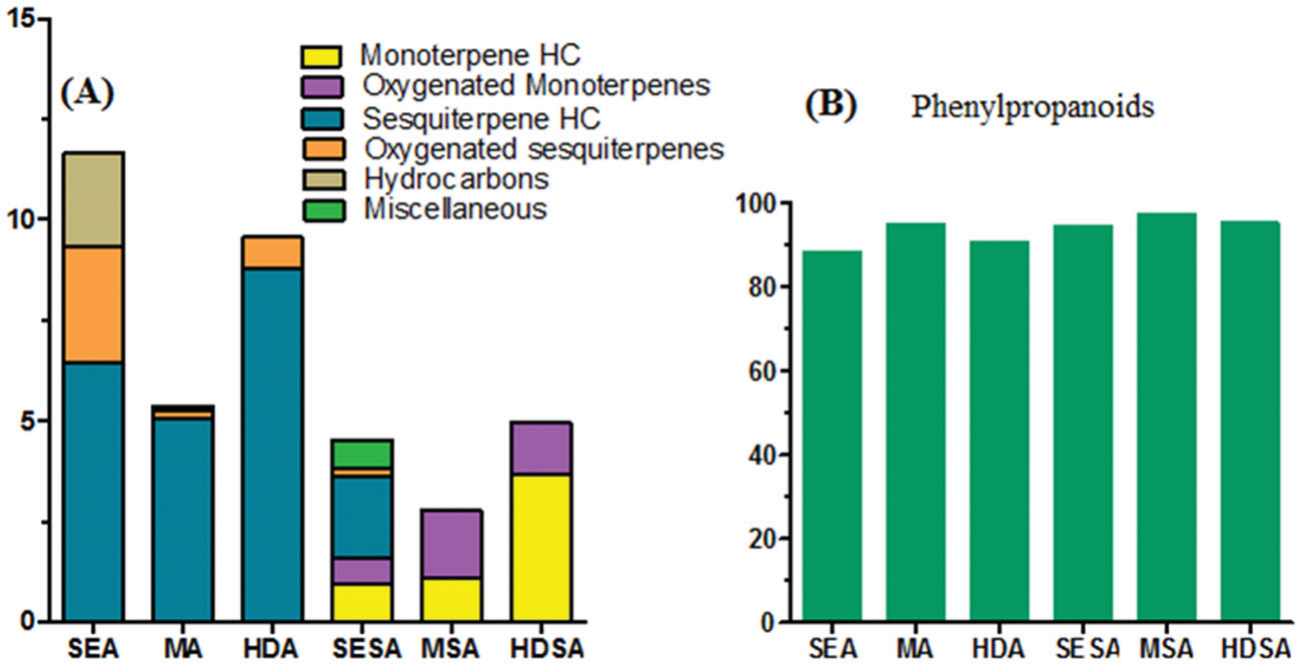
A schematic stacked bar charts showing relative percentages of total identified peak area (A) different classes of compounds except for phenyl propanoids and (B) phenyl propanoids class identified by GC-MS in anise and star anise using different extraction techniques.

In anise EO prepared by solvent extraction (SE), microwave-assisted extraction (MAE) and hydro-distillation (HD) techniques, other classes were predicated mainly on sesquiterpenes class *viz.* sesquiterpenes hydrocarbons (6.47, 5.06 and 8.80%, respectively) and oxygenated sesquiterpenes (2.87, 0.19 and 0.75%, respectively) in addition to hydrocarbons detected only in solvent extraction method (2.30%). In star anise EO prepared by SE, MAE and HD, the other classes identified were predominantly monoterpenes as monoterpene hydrocarbons (0.95, 1.09, 3.69%, respectively) and oxygenated monoterpenes (0.62, 1.68, 1.28%, respectively). The detected chemical classes and their relative percentages are simplified in Figure 2. All volatiles identified in both anise and star anise EO were summarised in Table 1.

**Table 1.** GC-MS analyses of volatiles of anise and star anise oils prepared by different methods (solvent extraction, microwave and hydrodistillation), whereas *n* = 3.

Peak no.	RT (min)	KI (obs.)	KI (lit.)	Compound name	% Composition (Mean of 3 independent runs ± SD)						
					Anise			Star anise			
					SEA	MA	HDA	SESA	MSA	HDSA	Identification
1	7.34	916	917	α-Pinene	–	–	–	–	–	0.396 **±** 0.03	MS, KI
2	9.63	999	1004	3-Carene	–	–	–	–	–	0.09 **±** 0.01	MS, KI
**3**	**10.05**	**1018**	**1018**	**D-Limonene**	**-**	**-**	**-**	**0.95 ± 0.05**	**1.09 ± 0.09**	**3.21 ± 0.09**	MS, KI
4	10.27	1020	1023	Eucalyptol	–	–	–	–	0.45 ± 0.04	0.20 **±** 0.03	MS, KI
5	12.47	1090	1082	Linalool	–	–	–	0.43 **±** 0.04	0.63 ± 0.10	0.78 **±** 0.16	MS, KI
6	14.87	1168	1177	terpinene 4-ol	–	–	–	0.04 **±** 0.01	0.15 ± 0.03	0.23 ± 0.02	MS, KI
7	15.27	1181	1185	α-terpineol	–	–	–	0.15 **±** 0.03	0.10 **±** 0.00	0.07 **±** 0.02	MS, KI
**8**	**15.51**	**1189**	**1195**	**Estragole**	**0.16 ± 0.08**	**-**	**-**	**1.57 ± 0.13**	**3.44 ± 0.12**	**1.63 ± 0.35**	MS, KI
9	17.29	1250	1251	*P*-anisaldehyde	–	–	–	1.69 **±** 0.19	–	–	MS, KI
10	17.18	1246	1245	*Cis*-anethole	–	–	–	0.13 **±** 0.02	–	–	MS, KI
**11**	**18.25**	**1283**	**1283**	***t*-Anethole**	**82.91 ± 0.59**	**93.21 ± 2.61**	**87.35 ± 0.22**	**82.48 ± 1.01**	**93.78 ± 0.42**	**93.39 ± 1.34**	MS, KI
12	19.52	1327	1337	δ-Elemene	0.05 **±** 0.01	0.06 **±** 0.01	0.31 **±** 0.01	–	–	–	MS, KI
13	20.62	1366	1376	α-Copaene	–	–	–	0.15 **±** 0.00	–	–	MS, KI
14	20.95	1378	1381	Anisketone	–	–	–	0.57 **±** 0.01	–	–	MS, KI
15	21.49	1379	1383	*Cis*-caryophyllene	–	–	–	0.23 **±** 0.01	–	–	MS, KI
16	21.67	1403	1405	*Trans*-α-bergamotene	–	–	–	0.96 **±** 0.02	–	–	MS, KI
17	22.82	1448	1446	β-Sesquiphellandrene	–	–	–	0.07 **±** 0.00	–	–	MS, KI
18	22.62	1440	1447	α-Himachalene	0.42 **±** 0.00	0.30 **±** 0.06	0.46 **±** 0.02	–	–	–	MS, KI
**19**	**23.4**	**1470**	**1470**	**γ-Himachalene**	**4.80 ± 0.01**	**3.54 ± 1.01**	**5.99 ± 0.11**	**-**	**-**	**-**	MS, KI
20	23.52	1475	1467	*trans*-caryophllene	0.24 **±** 0.01	0.15 **±** 0.03	–	0.30 **±** 0.01	–	–	MS, KI
21	23.76	1485	1495	Zingiberene	0.41 **±** 0.01	0.48 **±** 0.09	1.25 **±** 0.12	–	–	–	MS, KI
22	23.97	1493	1499	β-Himachalene	0.28 **±** 0.03	0.17 **±** 0.05	0.40 **±** 0.03	–	–	–	MS, KI
23	24.12	1499	1500	β-Bisabolene	0.27 **±** 0.01	0.36 **±** 0.00	0.39 **±** 0.01	0.35 **±** 0.01	–	–	MS, KI
24	24.22	1503	1668	unidentified	–	–	–	0.13 **±** 0.01	–	–	MS, KI
25	25.47	1551	1554	Nerolidol	–	–	–	0.10 **±** 0.08	–	–	MS, KI
26	27.80	1647	1580	α-cadinol	–	–	–	0.07 **±** 0.01	–	–	MS, KI
27	31.89	1828	2028	unidentified	–	–	–	0.05 **±** 0.01	–	–	MS, KI
**28**	**31.92**	**1830**	**1822**	**Pseudoisoeugenol 2- methyl butanoate**	**4.81 ± 0.08**	**1.42 ± 0.15**	**3.10 ± 0.08**	**-**	**-**	**-**	MS, KI
29	33.13	1887	1882	Thellungianin- G	2.87 **±** 0.08	0.19 **±** 0.03	0.75 **±** 0.06	–	–	–	MS, KI
**30**	**38.67**	**1670**	**1677**	**Foeniculin**	**-**	**-**	**-**	**7.41 ± 0.98**	**-**	**-**	MS, KI
31	40.75	2254	2467	Verimol H	0.37 **±** 0.03	–	–	0.13 **±** 0.01	–	–	MS, KI
32	42.85	2403	2394	Unidentified	–	–	–	0.22 **±** 0.01	–	–	MS, KI
33	43.53	2414	2286	Verimol C	–	–	–	0.26 **±** 0.01	–	–	MS, KI
34	44.13	2486	2358	Bemotrizinol	–	–	–	0.27 **±** 0.01	–	–	MS, KI
35	44.84	2531	2704	Unidentified	–	–	–	0.26 **±** 0.03	–	–	MS, KI
36	46.99	2670	2700	Heptacosane	0.65 **±** 0.08	–	–	–	–	–	MS, KI
37	49.89	2850	2900	Nonacosane	0.99 **±** 0.06	–	–	–	–	–	MS, KI
38	52.58	3029	3000	Triacontane	0.66 **±** 0.03	–	–	–	–	–	MS, KI
39	52.97	3055	3187	β-sitosterol	–	0.12 **±** 0.01	–	0.08 **±** 0.03	–	–	MS, KI
40	56.43	3276	3290	γ-sitosterol	–	–	–	0.36 **±** 0.06	–	–	MS, KI
**Monoterpene hydrocarbons**					**0**	**0**	**0**	**0.95**	**1.09**	**3.69**	
**Oxygenated monoterpenes**					**0**	**0**	**0**	**0.62**	**1.68**	**1.28**	
**Sesquiterpene hydrocarbons**					**6.47**	**5.06**	**8.80**	**2.06**	**0**	**0**	
**Oxygenated sesquiterpenes**					**2.87**	**0.19**	**0.75**	**0.17**	**0**	**0**	
**Phenyl propanoids**					**88.25**	**94.63**	**90.45**	**94.24**	**97.22**	**95.02**	
**Hydrocarbons**					**2.30**	**0**	**0**	**0**	**0**	**0**	
**Miscellaneous**					**0**	**0.12**	**0**	**0.71**	**0**	**0**	
**% Identified of total peaks area**					**99.89**	**100.00**	**100.00**	**98.75**	**99.99**	**99.99**	

The identified compounds are listed as per their retention time on column RTX-5 GC. **RT**: retention time (min), **KI (obs.)**: Kovat index observed experimentally on RTX-5 column compared to *n*-alkanes C_8_–C_28_**. KI. (Lit.):** Kovat index reported from previous data. All identified volatiles referenced to identification based on the comparison of their mass spectral data and KI to those published retention indices in the NIST (National Institute of Standards and Technology) library, adams and other literature data. **SEA**: solvent extraction anise; **SESA**: solvent extraction star anise; **MA**: microwave anise; **MSA**: microwave star anise; **HDA**: hydro-distillation anise; **HDSA**: hydro-distillation star anise. Bold values are the major constituents in each oil sample. All GC-MS runs for anise and star anise oils extracted by three extraction methods were assessed in independent triplets (Each GC-MS run for one replicate of each extraction method).

D-limonene, the major monoterpene detected in citrus fruits^31^ was only detected exclusively in star anise oil, with a higher percentage using (3.21%) using the hydrodistillation method. The sesquiterpene, γ-himachalene, was enriched only in the hydrodistilled anise oil (up to 5.99%), still not detected in star anise oil. The results of this current study were consistent with that reported by Gholivand et al.^6^, where they described the presence of *trans-*anethole, limonene, chavicol, and anisaldehyde as major components in star anise oil obtained by hydro-distillation–headspace solvent microextraction (HD-HSME) technique. However, in this current study of anise oil samples, chavicol was never detected by any of the three different extraction methods implemented so far. The development of an ultra-fast GC electronic nose coupled with a chemical method was described by Nie et al.^32^ to identify star anise oil constituents, which showed the presence of anethole, limonene, α-terpinene, and α-phellandrene as major components. The latter two were not detected in the star anise oil sample of this study in any of the implemented extraction methods. Previous studies on anise oil revealed similar components to those reported in the current study, where either hydrodistillation or supercritical fluid extraction of anise oil revealed the majority of *trans*-anethole, γ-himachalene and *trans*-pseudoisoeugenyl 2-methylbutyrate by Orav et al. and Rodrigues et al., respectively^33^^,^^34^ and *trans*-anethole, fenchone and methyl chavicol by Singh et al.^35^. These variations in chemical composition even implementing the same methodology may be attributed to the method of cultivation, the season of harvesting and method of drying or handling^10^. Other researchers used different extraction methods such as steam distillation, cold-pressing and extraction using *n*-hexane, where all of them resulted in an anethole-rich oil or extract with variable content^36–38^.

Regarding star anise, implementing different extraction methodologies such as steam distillation, simultaneous distillation, solvent and supercritical fluid resulted in the identification of *trans*-anethole (70.61%–77.31%) and estragole (1.71%–5.15%)^39^^,^^40^, which came in accordance with our results except for a higher anethole content. Microwave extraction carried out by Cai et al. revealed the presence of oxygenated organic compounds as well^8^. *cis*-Anethole identified only by star anise solvent extraction in our study, was previously reported in hydrodistillation of star anise carried out by Singh et al.^41^

The predominance of *trans-*anethole, its isomer estragole, and γ-himachalene in the hydro-distilled anise oil has likewise been previously reported by Özcan et al.^7^. The differences noticed in the concentrations of estragole and γ-himachalene may be attributed to the variations in environmental factors and agricultural habits which might affect the oil's chemical composition^7^.

The phenolic ester, pseudoisoeugenol-2-methylbutanoate, was only detected in anise oil (up to 4.81%) obtained by solvent extraction. In a similar way, the phenolic ester, thellungianin G, was solely detected in anise oil (*ca.* 2.87%) obtained by *n*-hexane extraction. The aromatic ether, foeniculin, was identified exclusively in star anise oil (*ca.* 7.41%) obtained by solvent extraction.

Based on their percentage in the overall oil, in anise, the major metabolites were identified as *trans-*anethole, γ-himachalene, pseudoisoeugenol-2-methylbutanoate, and thellungianin G, whereas in star anise oil, the predominant compounds were *trans*-anethole, estragole, and foeniculin (only by solvent extraction).

### 3.2. Multivariate data analysis of anise and star anise oils

The chemical composition, as well as the qualitative and quantitative variability of the anise and star anise essential oils extracted by three different extraction techniques, were further studied. Successful analysis of metabolite data utilises multivariate data analysis (MVDA) to compare diversities among the datasets. Unsupervised machine learning methods, such as hierarchical cluster analysis (HCA) and principal component analysis (PCA) search for patterns and clusters in unlabelled data^42^^,^^43^.

**Figure 3.**
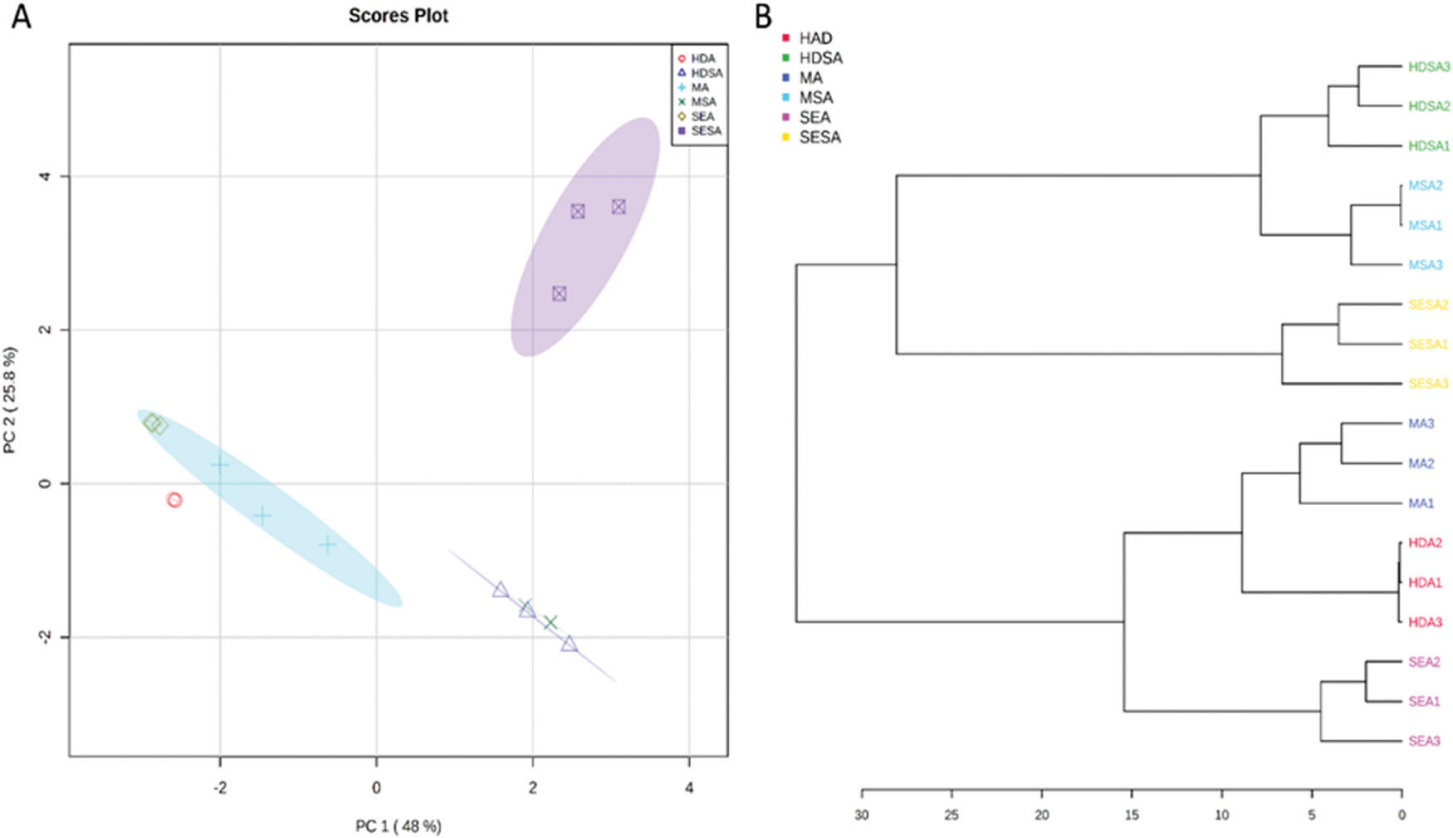
The score plot of principal component analysis (PCA) (A) and hierarchical clustering analysis (HCA) dendrogram (B) originated from the GC data set of anise and star anise samples obtained by different extraction methods.

Multivariate data analysis, as performed by PCA and HCA, revealed a clear separation of different extraction methods (Figure 3). PCA analysis revealed clear segregation of samples according to different extraction methods along with the first two principal components (PC1 and PC2), representing more than 70% of the total variance. Alongside the PC1, which accounts for 48% of the total variance, two major clusters of anise and star anise were segregated from each other. Regardless of the extraction method, anise and star anise samples were separated along the PC1. For anise essential oils, samples extracted by hydro-distillation were separated from those prepared by solvent or microwave-assisted extraction. While star anise oils showed clear segregation of samples prepared by solvent extraction from other samples along PC2, accounting for 25% of the total variance as in Figure 3(A).

Furthermore, to differentiate anise and star anise samples by extraction method, an unsupervised pattern recognition method (HCA) was implemented. A similarity measure was performed based on Euclidean distance using Ward's linkage clustering algorithms. HCA showed considerably similar results to those obtained by PCA for both the genotype and extraction methods. As shown in Figure 3(B), the samples were divided into two clusters in accordance with their species. The observed clustering pattern for the HCA tree was consistent with the PC1. According to the HCA tree, samples prepared by solvent extraction were distinctively excluded from other samples.

**Figure 4.**
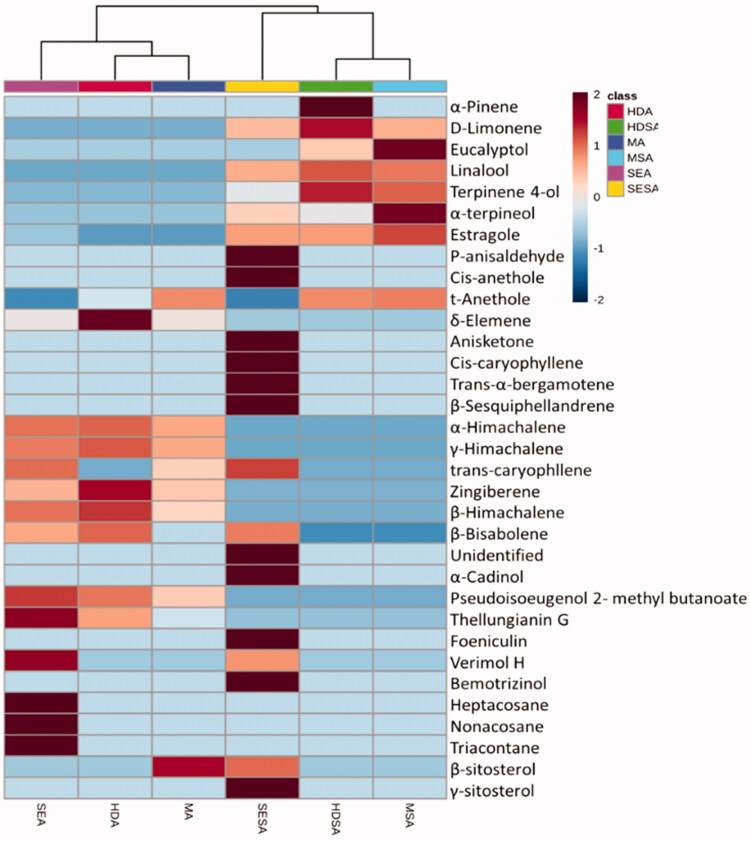
Heatmap represents the relative abundance of volatile metabolites extracted from anise and star anise fruits. Hierarchical clustering was done using Pearson’s correlation and average linkage. Heatmap colours represent log_10_ and Pareto-scaled values of relative metabolite abundance as indicated in the colour key. SEA: solvent extraction anise; MA: microwave extraction anise; HDA: hydro-distillation anise; SESA: solvent extraction star anise; MSA: microwave extraction star anise; HDSA: hydro-distillation star anise.

The chemical diversity of the oil samples from anise and star anise was also underlined by coloured bands exhibited by the heatmap shown in Figure 4. Heat maps are commonly used as a non-supervised visualisation tool, where the relative intensities of metabolites are represented with colour intensity through a scale. Bands with red colour show an increase in the relative abundance of metabolites and vice versa for blue bands. Heatmap clustering confirmed the results of the HCA analysis. The content of foeniculin, *p*-anisaldehyde, *cis*-anethole, *cis*-caryophyllene, *trans*-α-bergamotene and bemotrizinol led to the segregation of star anise oil prepared by solvent extraction. Thellungianin G, triacontane, heptacosane, nonacosane and verimol H contributed to anise oil prepared by solvent extraction.

### 3.3. Antioxidant activity in vitro using DPPH and ABTS assays

Star anise oil obtained by microwave extraction technique (MSA) enriched with the highest content of *trans*-anethole (93.78%) and D-limonene (3.21%) has been tested for its antioxidant activity *in vitro* using two different assays *viz.* DPPH and ABTS protocols. The results demonstrated promising antioxidant activity of star anise EO recording 9.43 ± 0.98 µM TE/mg star anise oil (IC_50_ 34.29 ± 0.77 mg/mL compared to Trolox 4.89 ± 0.26 µg/mL) and 8.23 ± 0.36 μM TE/mg star anise oil (IC_50_ 31.71 ± 0.19 mg/mL compared to Trolox 5.6 ± 0.12 µg/mL); respectively in DPPH and ABTS assays.

In a previous study by Destro et al., the antioxidant activity of star anise oil extracted by hydrodistillation using the Clevenger apparatus recorded better results than *trans*-anethole and D-limonene major constituents individually proving the synergistic action of star anise oil all constituents using DPPH (23.5 ± 0.3 mmol TE/g EO) and ABTS (25.7 ± 0.8 mmol TE/g EO) assays^44^. In a previous work by Aly et al.^45^, the recorded antioxidant activity of star anise oil extracted by hydrodistillation technique using DPPH assay was (55.6 mg/mL) versus (34.29 mg/mL) in this current study using the microwave. It could be correlated to the method of extraction where the microwave technique used in this current study revealed the enrichment of the star anise EO with *trans*-anethole (93.78%) and D-limonene (3.21%), whereas in previous work by Aly et al.^45^, star anise oil was prepared by hydrodistillation revealed *trans*-anethole and D-limonene percentile 82.7 and 2.3%; respectively^45^.

### 3.4. *In silico* study of star anise volatiles on human tyrosinase and NAD(P)H oxidase

**Figure 5.**
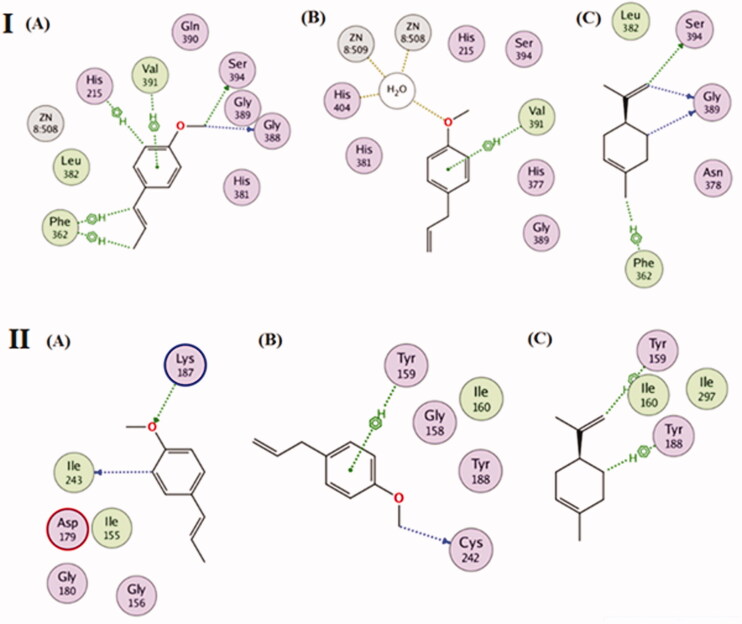
(I) 2 D-Binding diagram of *trans-*anethole (A), estragole (B) and D-limonene (C) with the active sites of tyrosinase enzyme. (II) 2 D-Binding diagram of *trans*-anethole (A), estragole (B) and D-limonene (C) with the active sites of NAD(P)H oxidase enzyme.

Previous studies revealed that the antioxidant activity of *I. verum* fruit essential oil was purported to the presence of *trans*-anethole, particularly due to its unsaturation^46^. Also, previous studies worked on understanding the influence of phenyl propanoids being antioxidants through *in-silico* enzyme inhibition studies on NAD(P)H oxidase and tyrosinase enzymes^47^^,^^48^. It was encouraging to test for the major components of star anise oil extracted by microwave technique exemplified by *trans*-anethole phenyl propanoid volatile on the inhibition of the previously mentioned enzymes *in-silico*. The *in-silico* study on both NAD(P)H oxidase and tyrosinase enzymes was conducted to investigate the possible mechanism of action in which the three major compounds *viz. trans*-anethole, estragole and D-limonene exert their antioxidant effect. The human tyrosinase and NAD(P)H oxidase play essential roles in oxidative stress, accordingly, the 3 D structures of the two enzymes were downloaded from the protein data bank using the following PDB IDs: 5m8q and 2cdu for human tyrosinase and NAD(P)H Oxidase, respectively. After that, the three major compounds were docked into the active site vicinity of both the enzymes. Interestingly, the three compounds achieved acceptable binding scores upon docking with the two targets. *Trans*-anethole achieved the highest docking scores, −8.9 and −10.1 Kcal/mole with human tyrosinase and NAD(P)H Oxidase, respectively. Estragole and D-limonene achieved lesser scores than trans-anethole, exactly −8.2 and −7.2 Kcal/Mole with human tyrosinase and −8.8 and −7.6 with NAD(P)H Oxidase, respectively. As depicted in Figure 5(I), *trans*-Anethole was able to interact with His215, Phe362, Gly388, Val391 and Ser394 in the active site of human tyrosinase. Similarly, estragole bonded with human tyrosinase through interacting with Phe362 and Zinc508, Zinc509 and His404 through a solvent bridge contact, on the other hand, D-limonene interacted with Phe362, Gly389 and Ser394 residues of human tyrosinase. Figure 5(II) highlights the interactions of the three components with NAD(P)H oxidase in which *trans*-anethole formed two interactions with Lys187 and Ile243, Estragole interacted with Tyr159 and Cys242, while D-limonene was able to interact with Tyr159 and Tyr188. The docking study conducted on star anise's main constituents, mainly *trans*-anethole followed by estragole and D-limonene, were proved as potential antioxidants by inhibiting the human tyrosinase and NAD(P)H oxidase.

### 3.5. Cytotoxicity screening of *trans*-anethole enriched oil

Since the oils extracted by the MAE technique showed the major existence of *trans-*anethole in both anise and star anise, with a higher percentile in star anise volatile oil, the latter was assessed for its cytotoxic activity versus variable cancer cells lines including hepatocellular carcinoma (HepG2), ovarian (SKOV-3), breast (MCF-7), cervical (HeLa), and prostate (PC-3) cancer cell lines. The results revealed promising cytotoxic activity recording IC_50_ values of 0.19 ± 0.019 (comparable to doxorubicin, with IC_50_ of 1 µg/ml), 0.18 ± 0.008 (comparable to doxorubicin, with IC_50_ of 0.2 µg/ml), 0.19 ± 0.011 (comparable to doxorubicin, with IC_50_ of 0.2 µg/ml), 0.16 ± 0.005 and 0.28 ± 0.004 mg/mL; respectively.

**Figure 6.**
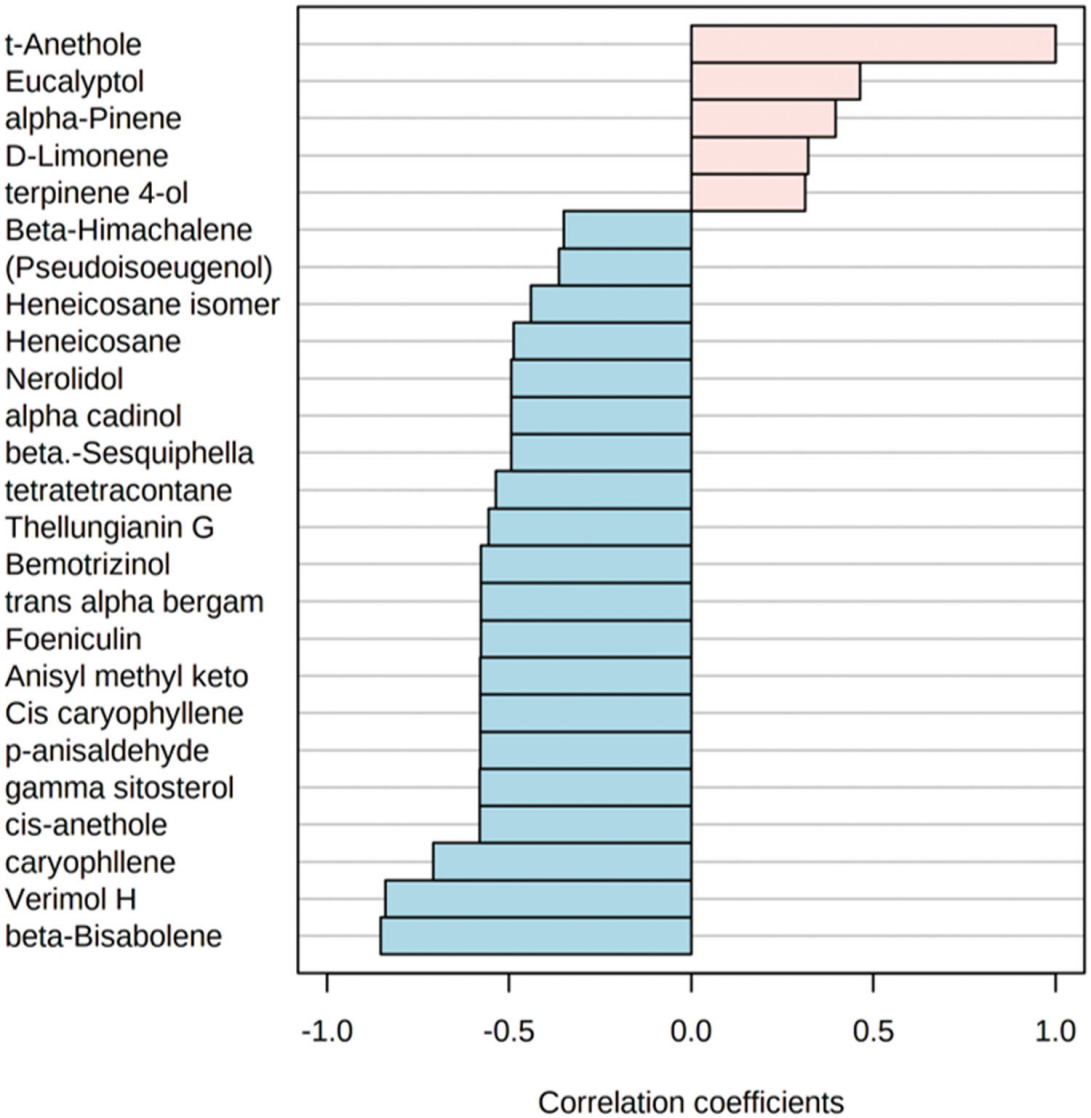
Correlation coefficients of different volatiles correlated to *trans*-anethole.

Upon utilising the multivariate data analysis to correlate the volatile components of the oils and *trans*-anethole, the major component. It could be concluded from Figure 6 that there is a positive correlation between volatiles such as eucalyptol, α-pinene, D-limonene and terpinene-4-ol with *trans*-anethole. It could be concluded from the GC-MS analysis Table 1 of the star anise oil extracted by microwave that not only *trans*-anethole is the chief volatile effective as a cytotoxic agent but also D-limonene (1.09%) followed by eucalyptol (0.45%) and terpinene-4-ol (0.15%) could influence the cytotoxicity study on different tested cell lines as well.

Chen and de Graffenried, proved the effectiveness of *trans*-anethole as an anti-cancer agent on MCF-7 and MDA-MB-231 oestrogen-dependent breast cancer cell lines through modulation of cell survival, proliferation as well as apoptosis^49^. In another study, essential oil of star anise was tested on different cancer cell lines whereas the best IC_50_ recorded on colon cancer cell line (HCT-116) was 50.34 ± 1.19 μg/mL, whereas the major identified volatile component was *trans-*anethole. The mechanism of the cytotoxic activity of natural products was studied in many previous literature data^50^^,^^51^. Asif et al. correlated the cytotoxicity mechanism to morphological changes in the cell nucleus, the negative potential on the mitochondrial membrane in a dose-dependent manner in addition to cell migration inhibition, invasion, and formation of colonies in the treated cells with star anise oil^52^. It is worth to mention estragole content in star anise was enriched in the oil prepared by the MAE technique (3.44%) followed by hydro-distillation (1.63%) and then solvent extraction (1.57%). In previous work, fennel seeds volatile oil with estragole content (3.89%) recorded a dose-dependent apoptosis action accompanied by cell cycle arrest in the G2/M phase on the HepG2 cell line using MTT assay^53^.

### 3.6. Cell cycle distribution and annexin V-FITC apoptosis assay on HeLa cell line

Further investigations were performed on the cytotoxicity mechanistic role of anethole-rich star anise-rich oil on HeLa cancer cells. Cell cycle distribution followed by apoptotic assessment of HeLa cells after treatment (48 h) by anethole-enriched oil (MSA) was performed using flow cytometry.

#### 3.6.1. Cell Cycle distribution

**Figure 7.**
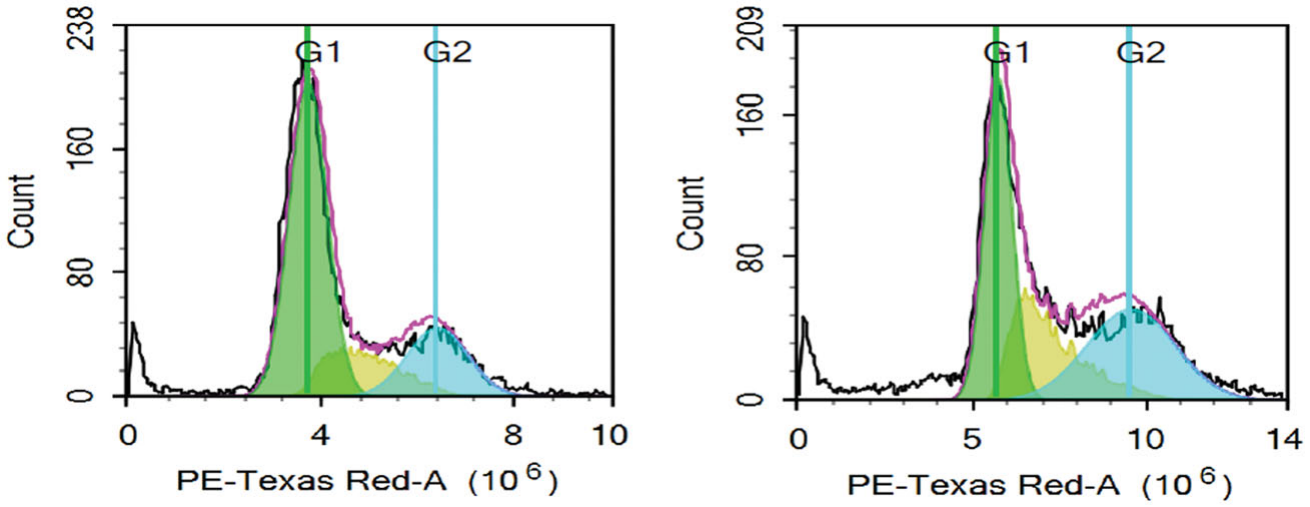
The effect of *trans*-anethole enriched Star anise oil on the phases of the cell cycle of HeLa cells.

In this work, HeLa cells were treated with MSA oil to investigate its effect on the cell cycle. As Table 2 summarises, at the G0–G1 phase the MSA oil was able to reduce the cells population to 37.19% as compared with the control (58.71%). In addition, the oil resulted in a noticeable increase in the cell population at S and G2/M phases, from 15.79% and 19.47%, respectively to 23.09% and 29.96%, respectively. Regarding the sub-G1 phase, a slight increase was observed in the percentage of the cells from 5.26% (control cells) to 9.76% in the cells treated with the oil (Figure 7, Table 2). Accordingly, we could conclude that the exerted cytotoxic effect of the oil is attributed to cell cycle inhibition at the G0–G1 phase.

**Table 2.** The effect of *trans*-anethole enriched Star anise oil on the phases of the cell cycle of HeLa cells.

	% G0–G1	%S	% G2/M	%Sub-G1
MSA oil	37.19	23.09	29.96	9.76
Paclitaxel	58.71	15.79	19.47	5.26

#### 3.6.2. Annexin V-FITC apoptosis assay using flow cytometry

**Figure 8.**
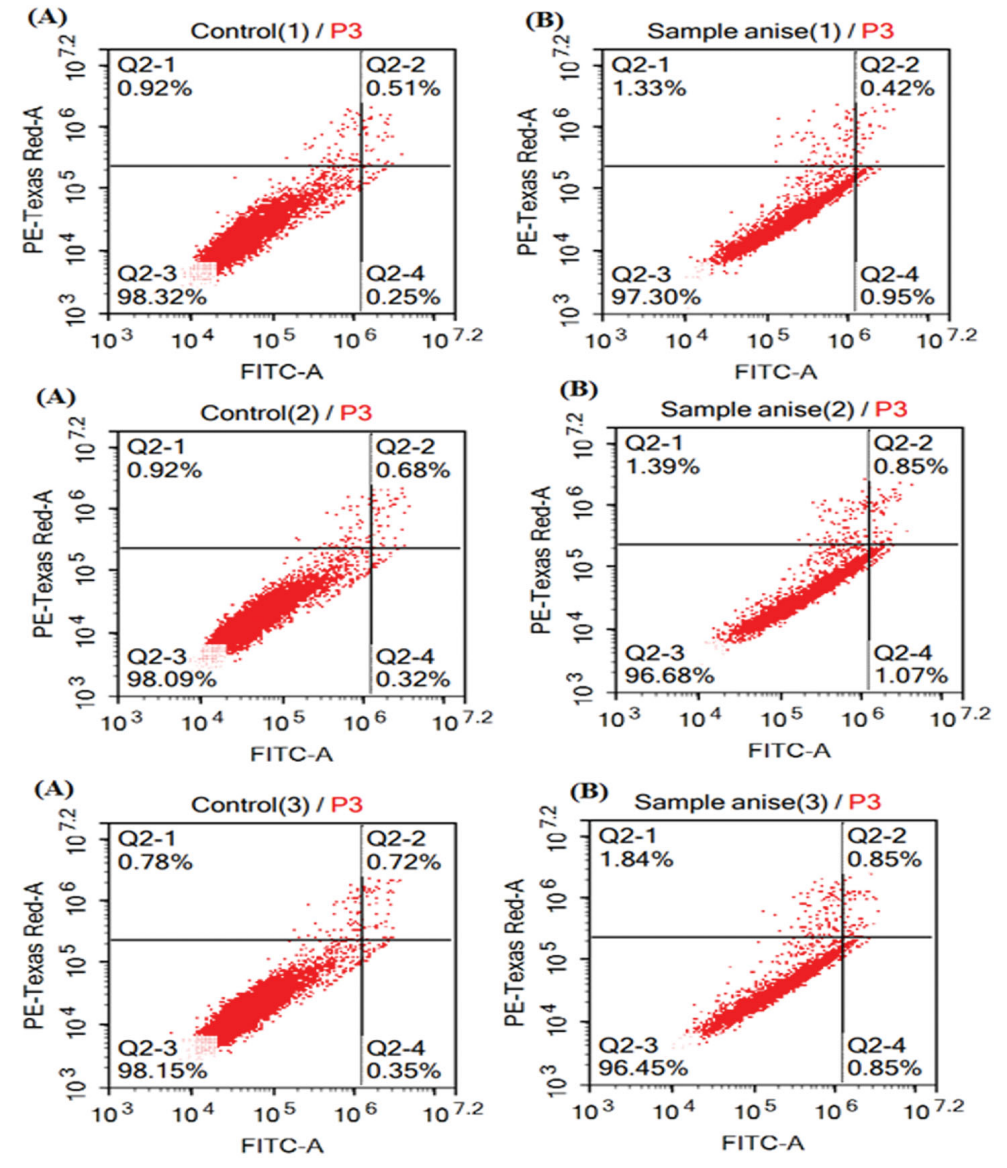
Triplicate Annexin V-FITC apoptosis assay for *trans*-anethole enriched Star anise oil (MSA) (B) using flow cytometry as compared to Doxorubicin control (A).

The apoptotic effect of MSA oil was evaluated using Annexin V-FITC apoptosis detection kit. Investigating the results revealed that the oil increases the total apoptosis to 1.68% (Lower Right = 0.97%, Upper Right = 0.71%), in comparison to the control cells that showed a total percentage of apoptosis equals 0.95% (Lower Right = 0.31%, Upper Right = 0.64%) (Figure 8, Table 3). Accordingly, the results suggest an early apoptotic effect for the oil in HeLa cells.

**Table 3.** The results of the Annexin V-FITC apoptosis assay for *trans*-anethole enriched Star anise oil (MSA).

	Early apoptosis (Lower Right %)	Late Apoptosis (Upper Right %)	Total (L.R % + U.R %)
MSA oil	0.97	0.71	1.68
Doxorubicin	0.31	0.64	0.95

### 3.7. *In silico* study of star anise volatiles on EGFR, CDK2 and CDK4

**Figure 9.**
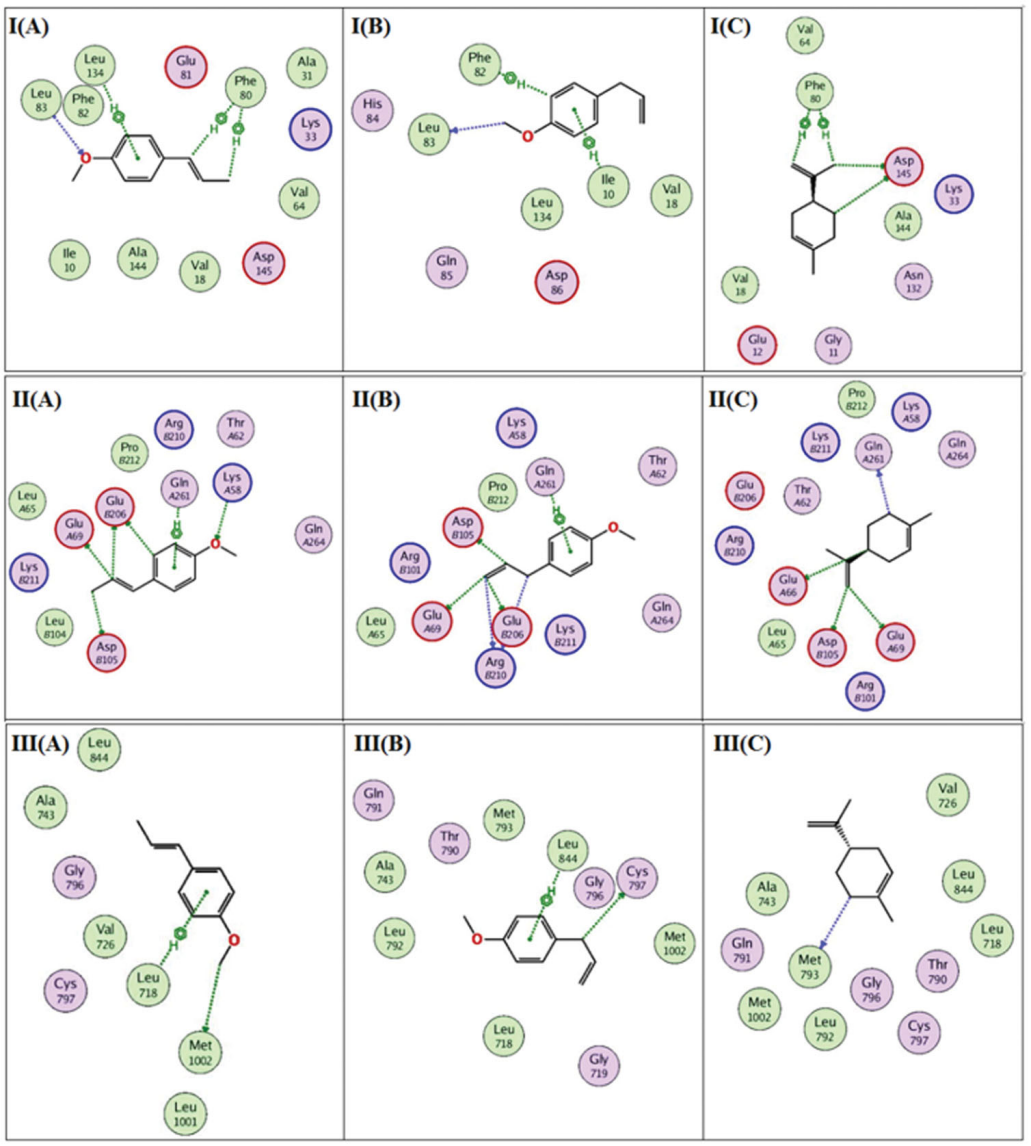
2 D-Binding diagram of *trans*-anethole (A), estragole (B) and D-limonene (C) with the active sites of CDK2 (I), CDK4 (II) or EGFR (III).

This part was conducted to investigate the possible mechanism of action in which the three major compounds *viz. trans*-anethole, estragole and D-limonene exert their cytotoxic effect. In addition, this part was also conducted to provide extra insights for further future experimental assays. EGFR, CDK2 and CDK4 play essential oncogenic roles in many types of cancers. Accordingly, the 3 D structures of the three enzymes were downloaded from the protein data bank using the following PDB IDs: 1xkk, 1di8 and 2w96, respectively. After that, the three major compounds were docked into the active site vicinity of the three enzymes EGFR, CDK2 and CDK4. Interestingly, the three compounds achieved acceptable binding scores upon docking with the three targets. *Trans*-anethole achieved the highest docking scores, −7.9, −9.3 and −9.9 Kcal/mole with EGFR, CDK2 and CDK4, respectively. Estragole and D-limonene achieved lesser scores than *trans*-anethole, exactly −7.1 and −6.4 Kcal/Mole with EGFR, −7.9 and −7.5 Kcal/Mole with CDK2 and −8.3 and −7.9 Kcal/Mole with CDK4, respectively. As depicted in Figure 9, *trans*-anethole was able to interact with Leu718 and Met1002 in the active site of EGFR. Similarly, Estragole bonded with EGFR through interacting with Cys797 and Leu844on the other hand, D-limonene interacted with Met793 residue of EGFR. Figure 9 highlights the interactions of the three compounds with CDK2 in which *trans*-anethole formed four interactions with Phe80, Leu83 and Leu134, Estragole interacted with Phe82, Leu83 and Ile10, while D-limonene was able to interact with Phe80 and Asp145. Investigating Figure 9, *trans*-snethole was able to interact with Lys58, Glu69, Asp105, Glu206 and Gln261 in the active site of CDK4. Similarly, Estragole bonded with CDK4 through interacting with Glu69, Asp105, Glu206, Arg210 and Gln261 on the other hand, D-limonene interacted with Glu66, Glu69, Asp105 and Gln261 residues of CDK4. The docking study conducted on star anise main constituents, mainly *trans*-anethole followed by estragole and D-limonene, were proved as potential cytotoxic agents through inhibiting EGFR, CDK2 and CDK4. In addition, the docking results guided us to perform further apoptosis and cell cycle studies.

## 4. Conclusion

Anise and star anise fruits oils were extracted by different techniques such as hydrodistillation, solvent (*n*-hexane) extraction and microwave-assisted extraction, then analysed by GC-MS to reveal the privilege of phenylpropanoids (88.25–97.22%) among all tested samples, containing mainly *trans-*anethole, for which results revealed the best extraction method to be assisted by microwave. A comparative chemometric analysis was performed via Principal Component Analysis (PCA) and Hierarchical Clustering Analysis (HCA), demonstrating a clear separation of different extraction methods. Anise hydrodistilled oil was separated from those prepared by other methods, and clear segregation of star anise samples prepared by solvent extraction from other samples was observed. Antioxidant activity *in-vitro* using DPPH and ABTS assays revealed values of 9.43 ± 0.98 µM TE/mg star anise oil (IC_50_ 34.29 ± 0.77 mg/mL) and 8.23 ± 0.36 μM TE/mg star anise oil (IC_50_ 31.71 ± 0.19 mg/mL); respectively. Furthermore, the docking endorsed the ability of the three major compounds *viz. trans*-anethole, estragole and D-limonene as potential antioxidants by inhibiting the human tyrosinase and NAD(P)H Oxidase, especially *trans*-anethole. Cytotoxicity screening performed on anethole-rich star anise oil, revealed promising cytotoxic activity against various cell lines. Further investigation on the cell cycle analysis distribution and apoptosis assay was conducted on the HeLa cell line treated with anethole-rich oil to understand the mechanistic role of the oil being an effective anticancer agent. An *in silico* study on three main enzymes EGFR, CDK2 and CDK4 proved the effectiveness of *trans*-anethole in inhibiting these enzymes and so the consequences of cancer progression. The current study results can be of great importance in demonstrating biological activities and quality control analyses of anise and star anise.
